# Research Progress and the Development Trend of the Utilization of Crop Straw Biomass Resources in China

**DOI:** 10.3389/fchem.2022.904660

**Published:** 2022-05-12

**Authors:** Wei Yang, Xiaohua Li, Yutao Zhang

**Affiliations:** ^1^ College of Resources and Environmental Engineering, Anshun University, Anshun, China; ^2^ University Rural Revitalization Research Center in Guizhou, Anshun, China; ^3^ College of Chemistry and Chemical Engineering, Anshun University, Anshun, China

**Keywords:** crop straw biomass, resource utilization, progress, development trend, CiteSpace

## Abstract

The utilization of crop straw biomass resources is highly emphasized by governments and academia in recent decades. Based on the core databases of the literature in China National Knowledge Infrastructure (CNKI) academic journals, CiteSpace software is used to analyze and process the hotspots, and this study proposed the primary coverage and evolutionary trends of research on the utilization of crop straw resources. The thesis proposes the research development trend for improving the institutional mechanism of the utilization of crop straw resources, strengthening technology research and development, exploring the economic model of green cycle agriculture, accelerating the construction of the industrial system, and designing new paths of resource utilization in multiple ways, which helps estimate the development trend of the utilization of crop straw resources and provide inspiration and direction for future research and practices.

## Introduction

The effective utilization of crop straw biomass is an important basis for the development of green agriculture and directly affects the construction of the rural ecological environment (Nie, 2021). It is a significant topic of common concern about how to efficiently use and scientifically treat it in all countries nowadays, and academics have carried out multifaceted research on this problem at home and abroad. Developed agricultural countries attach great importance to the resource utilization of straw and have carried out a series of studies on the usage methods (Champagne, 2008; Arthur and Baidoo, 2011). For example, in the United States, 45 million tons of wheat straw was utilized each year. Special funds were set up by the Department of Agriculture and Energy to research biomass fuels such as straw, and they have built 16 ethanol refineries, and certain government subsidies were given to them (Zhu and Yu, 2012). In Japan, 75% of straw is returned to the field every year or processed into roughage for cattle and sheep. The Japanese government pays great attention to developing and upgrading the straw utilization technology (Liu, 2014), and the law stipulates that straw must be recycled (Jia, 2015). In Denmark, the development of direct-fired biomass power generation is outstanding. They have built 13 straw power plants, and straw power generation accounts for 81% of the total renewable energy in the country (Fu, 2007). In Europe and the US, the way and amount of crop straw returned are clearly defined in the law in order to promote the standardization of crop straw utilization (Zhu, 2014), and they achieved good performance. For example, in Canada, about 67% and 73% of crop straw are recycled in Canada and England, respectively (Zhu, 2014). China is a large agricultural country with more than 1.4 billion people and a country with large straw production each year. Data show that China produced about 700 million tons of crop straw in 2012 (Chen et al., 2012), accounting for 18% of total agricultural organic waste (Zhou et al., 2017), and the number increased to 827 million tons in 2017 (Cong et al., 2019). With the increase in huge pressure of straw biomass disposal, people are increasingly concerned about this issue (Chen and Shi, 2017). China's government attaches great importance to the utilization of crop straw resources. Domestic research on straw resource utilization is slightly delayed compared to other developed countries, and over the years, the governments have issued a series of documents to prohibit straw burning and promote diversified utilization pathways to accelerate straw utilization. However, squander of straw also can be observed sometimes for economic benefits and labor costs (Wen et al., 2018). Research results show that China’s crop straw is utilized in various ways, and it is used to generate energy (Liu et al., 2020), forage (Duan, 2018), fertilizer (Ma and Dong, 2020), raw material, and base material (Wu et al., 2010). The main approach is to return the straw to the field (Bi et al., 2009). In 2008, straw harvested from 26.76 million hm^2^ was returned to the field (Bi, 2010), but the utilization of straw resources, especially the industrial development, is still limited. The technical system is still immature, and a series of problems need to be resolved (Liu et al., 2011). Therefore, a systematic analysis of crop straw resource utilization hotspots, content, and the current situation can help in studying and judging the development trend of straw resource utilization and provide inspiration and direction for future research and practice.

Based on the CNKI database, core journal papers database, using CiteSpace software, and graphical compilation, the authors mapped and analyzed the progress and trends of crop straw resource utilization research in China. The authors summarized and sorted out the main research hotspots to judge the future development trend of research on straw utilization.

## Data Sources and Research Methods

### Data Sources

The main subject of this article is the utilization of crop straw resources. The literature studies that the author has studied are core papers in the CNKI database, and these papers are indexed by the source journals from SCI, EI, core journal Peking University, CSSCI, and CSCD. The author set the search terms as *the utilization of crop straw resource*, *the utilization of crop straw feed*, *the utilization of crop straw energy*, *the utilization of raw material*, *the utilization of crop straw fertilizer*, *crop straw base utilization*, or *crop straw industrial utilization* with all years.

The index results showed that from 1994 to 2022, 135 articles studied the straw utilization from core journals in the CNKI database. The author removed four articles from the first online journals and eight articles from other volumes that were not part of the research papers. Eventually, 123 relevant literature studies from 2002 to 2021 were pitched to analyze and study.

### Research Methodology

CiteSpace software is commonly used in academic research because it enables readers to study the literature on a specific topic in scientific research in a visual way to obtain a scientific knowledge map in terms of layout structure, change pattern, publication time, subject area, author institution, and research level (Hu et al., 2022). In this study, CiteSpace software combined with fuzzy clustering analysis and correlation analysis is used to explore the research hotspots, main contents, evolutionary trends, and their correlations with the utilization of crop straw resources. The author analyzed the intrinsic connections of research hotspots at different stages and then studied the development direction of future research.

## Research Hotspots and Evolution Trends

### Research Hotspots

The keywords or subject terms can reflect the core content and research hot spots of the study perfectly (Shi and Tong, 2018). The authors analyzed the high-frequency keyword co-occurrence with CiteSpace software and summarized the hot spots of the research of crop resource utilization in each period. These keywords include *the utilization of crop straw fodder, biomass energy utilization, resource utilization, lignin degradation, biofermentation, straw gasification, base material utilization, industrial utilization, rational utilization, industrial chain, farmers’ willingness,* and *livestock carrying capacity*. In summary, they focused on high-frequency co-occurring words such as resource utilization, energy, forage utilization, fertilizer utilization, industrial raw material, base material utilization, and circular economy, reflecting the research hotspots of crop resource utilization in the last decade.

### Evolution Trends

**FIGURE 1:**
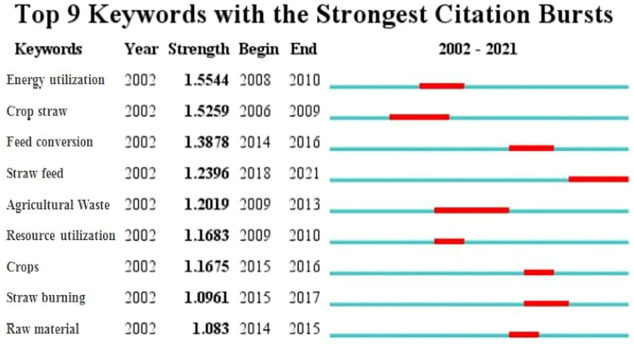
Top nine high-frequency emergent words.

The keywords or subject terms in the relevant literature were analyzed for emergent terms (Figure 1) to determine the development and new directions in the field of the research of crop straw resource utilization. The author used the time-zone function of CiteSpace software to explore the emergent terms to determine the emerging trends of research topics. Figure 1 shows the top nine emergent terms in the last 10 years: the first one is energy utilization with 1.5544 intensity from 2008 to 2010. The second one is straw fertilization with 1.5259 intensity from 2006 to 2009. The following terms are fodderization from 2014 to 2016, straw fodder from 2018 to 2021, agricultural waste from 2009 to 2013, resourcization from 2009 to 2010, crops from 2015 to 2016, straw burning from 2014 to 2015, and raw material from 2014 to 2015. The intensity of straw burning and straw raw materialization is the lowest. Straw burning causes severe environmental pollution, and how to reduce pollution induced by CO_2_ emissions and make straw into a propellant for ecological civilization and ecological environment construction is still a problem to be solved.

At present, straw is used as raw materials for industrial and agricultural utilization. Agricultural utilization mainly includes being used as the planting substrate of edible fungi and flowers. However, this utilization is at a relatively low level at this stage with immature technology and low effectiveness, so there are still many issues to be further explored.

### Main Research Content

Based on the analysis of the occurrence time and frequency of keywords, the main research contents of agricultural straw resource utilization were divided into five utilization approaches (Table 1).

1) Fertilization of Straw

**TABLE 1:** Pathway of crop straw resource utilization.

Entry	Utilization Approach	Technical Connotations	Application Area	Evaluation	Reference
1	Utilization as fertilizers	Straw is decomposed into an organic fertilizer or returned to the field directly.	Organic fertilizer	Enhance soil fertility and increase yield. Ecological effects under different tillage conditions are unclear.	Ding et al. (2010)
2	Utilization as forage	Straw is used as fodder directly or silage into forage that can be easily digested by animals.	Forage for livestock	After physical, chemical, and biological processing, straw has a higher value, but the operation process is complicated.	Wang et al. (2009)
3	Utilization as energy generation material	Straw is burned directly or converted to heat, gas, electricity, and other forms of energy.	Used to generate biogas and power; liquefaction, solidification, and carbonization of straw	The technology is imperfect for low overall utilization, high cost, and secondary pollution.	Xiao et al. (2010)
4	Utilization as industrial raw materials	Straw is rich in cellulose and is processed as a raw material for industrial production.	Plate, paper, packaging materials, starch extraction, and production of thermal insulation material	Resource-saving and environment friendly with bright prospects	Fan and Zhou, (2004)
5	Utilization as base material	Straw is used as strain culture, seedling, and lawn substrate.	Substrate for edible fungi, flower seedling base material, and lawn base material	Drives the growth of straw utilization scale effectively.	Shi et al. (2016)

Returning straw to the field improves soil fertility well because crop straw is rich in organic matter and minerals (Yang et al., 2011), making good natural suitability and economic benefits (Chen et al., 2020). Crop straw can be returned to the field directly or indirectly. Direct return mainly includes burying rice or cornstalk in the field, mixing corn straw within the plow layer, mulching corn in the field, and rototilling rice straw in the field (Han and Zou, 2018). Indirect return involves crop straw composting (Zhang et al., 2016), making biogas and its by-product fertilizer utilization (Zhao et al., 2017), combining straw utilization with farming (Shi, 2018), straw carbonization (Wu et al., 2015), straw substrate fertilization, and other technical models (Zhang, 2017), while indirect return to the farmland is faced with difficulties such as cost increase caused by the input of agricultural implements, machinery and labor, difficulty in storage, and pollution in the fermentation process.

2) The Straw Utilization of Forage

The utilization of straw for feed, especially studying the development of the food-saving livestock industry and promoting China’s food security guarantee, is an important measure of sustainable agricultural development. Straw feed processing methods mainly include straw bulking, ammonification, pellet feed, straw silage, straw compaction, and straw microstorage. The integration and optimization of the aforementioned technologies are effective means to promote the utilization of straw for feed. Technological progress is mainly achieved through the optimization of the main body, process, facilities, and auxiliary parts. (Qi et al., 2013).

3) The Straw Utilization of Energy

At present, straw energy utilization modes mainly include straw to biogas, straw centralized gas supply, straw power generation, and straw solidification molding (Li et al., 2010).

The utilization of biomass to generate energy is an important source of energy in the future. There are various ways of energy utilization, but the utilization technology needs to be further explored. At present, the main modes of straw energy utilization include biogas production, straw-fired cogeneration, and densified corn stover briquetting fuel (Li et al., 2010).

Practice shows that the efficiency of straw-fired cogeneration is low while the cost is high (Li et al., 2010). Currently, fuel enterprises in operation are mainly small- and medium-sized enterprises, which need to be improved in scale utilization and market recognition (Guo and Wand, 2017). The research shows that in the process of straw energy utilization, the cost of straw includes the input of raw material purchase, transportation, collection, and loading, which is generally high (Han and Zou, 2018). To promote straw energy utilization, many studies should be solved in the future to decrease the cost.

4) Straw Raw Materialization and Base Materialization Utilization

Straw is rich in lignocellulose and can be used as raw material for industrial products after a series of processes. This process can save resources and increase income at the same time. Raw materialization of straw is a process in which crop straw is transformed into lightweight synthetic panels, functional flooring, wooden doors or windows, and other construction materials for usage. Crop straw is used as a substrate generally for flowers, seedlings, and microbial strains (Li, 2014). Some scholars have concluded that straw materials can be used as building insulation, which has the advantage of reducing CO_2_ emissions and saving energy (Ma and Dong, 2010). However, the proportion of straw used as raw material for industrial products is still relatively small, and the technology of straw materialization needs to be improved to increase the scale of raw material utilization.

## Research Conclusions and Development Trends

### Conclusion of the Study

This study tries to estimate the utilization of crop straw resources in China by analyzing the research hotspot, research content, and the evolution trend of research direction using CiteSpace software. The results showed that the research frequency of crop straw resource utilization is on the rise, and the number of articles increased from 2002 to 2021, and the high-frequency co-occurring words with crop straw resource utilization mainly involve energy utilization, straw fertilization, straw fodder, straw resourcization, straw burning, and raw materialization, reflecting the research hotspot in different periods. In addition, the main content includes the straw utilization as fertilizer, forage, energy, raw materialization, and base materialization. Last, from the perspective of the evolution trend of research content, the research content gradually changes from increasing the utilization rate of straw resources to improving the level of the utilization technique.

### Development Trend

The implementation of a series of national strategies such as new countryside construction, beautiful countryside construction, territorial space planning and rural revitalization, efficient utilization of resources, ecological environment protection, and high-quality economic development has become the theme of China’s development. Based on the analysis of the current predicament and research progress of crop straw resource utilization, the authors assume that more attention should be paid to the following area:

1) The systems and mechanisms should be improved for using crop straw as resources. Under the leadership of the government, professional teams were organized to conduct in-depth research; the policy obstacles in the utilization of crop straw as resources were clarified, and targeted policies about financial support, technical assistance, incentive system, and performance assessment for the utilization of crop straw as resources were formulated.
2) Research on the resourcization technique of crop straw should be strengthened. The backward technology of straw recycling results in high production costs and small scale of straw-consuming enterprises. Strengthening the technical level of straw industrial utilization is the most important task at present.
3) It is necessary to adhere to the concept of carbon peak and carbon neutrality to explore the green circular agricultural economic model. Governments should promote the recycling of crop straws, especially the economical and intensive utilization of straw resources, and explore the suitable mode of green agriculture and recycling agriculture.
4) The construction of an industrial system should be accelerated for the utilization of crop straw resources. Countries all over the world, especially developed countries, should guide the exploration of diversified industrial utilization of crop straw, such as fertilizer, energy, feed, and raw material. The straw resource utilization industrial system should be constructed to get rid of crop straw resource utilization of small scale and low benefit predicament.
5) The utilization paths of crop straw are designed in diverse ways. In view of the needs of rural revitalization, beautiful countryside construction, and territorial space planning in the new era, a diversified design of the crop straw resource utilization path is carried out so as to explore a low-carbon and high-efficient regional straw resource utilization path tailored to local conditions.

The raw data supporting the conclusions of this manuscript will be made available by the authors, without undue reservation, to any qualified researcher.
